# Understanding scholar-trajectories across scientific periodicals

**DOI:** 10.1038/s41598-024-54693-7

**Published:** 2024-03-04

**Authors:** Yangliu Fan, Anders Blok, Sune Lehmann

**Affiliations:** 1 Copenhagen Center for Social Data Science, University of Copenhagen, Copenhagen, Denmark; 2 Department of Sociology, University of Copenhagen, Copenhagen, Denmark; 3 DTU Compute, Technical University of Denmark, Lyngby, Denmark

**Keywords:** Engineering, Computational science, Scientific data

## Abstract

Despite the rapid growth in the number of scientific publications, our understanding of author publication trajectories remains limited. Here we propose an embedding-based framework for tracking author trajectories in a geometric space that leverages the information encoded in the publication sequences, namely the list of the consecutive publication venues for each scholar. Using the publication histories of approximately 30,000 social media researchers, we obtain a knowledge space that broadly captures essential information about periodicals as well as complex (inter-)disciplinary structures of science. Based on this space, we study academic success through the prism of movement across scientific periodicals. We use a measure from human mobility, the radius of gyration, to characterize individual scholars' trajectories. Results show that author mobility across periodicals negatively correlates with citations, suggesting that successful scholars tend to publish in a relatively proximal range of periodicals. Overall, our framework discovers intricate structures in large-scale sequential data and provides new ways to explore mobility and trajectory patterns.

## Introduction

Publications in scientific journals and conference proceedings serve as the primary mode of knowledge production and communication and have become important indicators of individual research output and success^1–5^. Given the many underlying factors that influence a researcher's publication venues, ranging from individual selection criteria and strategies to discipline- or environment-based priorities and restrictions^6–9^, researchers are likely to follow many diverse publishing patterns. A researcher's publication trajectory across *scientific periodicals*—referring here to journals and proceedings—over time reveals important information on the characteristic locus of individual scientific activity.

The publication trajectory over the course of a researcher's career has been studied in the literature, with a common focus on the performance patterns in terms of publication counts or received citations^1,10–13^. While these indicators (e.g., publications and citations) have been used as the most prominent basis for tracking the author's career profile, there have also been attempts to understand the publication trajectory that incorporates more fine-grained information, such as the research topic, author affiliation, collaborator, or bibliographic network^14–19^. For example, a recent study explores the mobility of researchers across research topics. They find a significant negative correlation between the author's mobility in the semantic space and academic success, measured through the *h*-index^15^.

In this article, we study publication trajectories, focusing on the author's mobility in the space of scientific periodicals, which we consider as the fine-grained subdivision of science. The space encodes how periodicals can be functionally substituted for one another when authors journey through science. We aim to understand academic success through the prism of movement across these periodicals. This approach provides unique insights into the dynamic nature of research careers, as each choice of the periodical in one's research history records a wealth of information, such as strategic decisions, fields of study, and research status. A search of the literature revealed no previous study investigating the author's positioning and mobility patterns across publication venues. To the best of our knowledge, this is the first empirical study that reconstructs scholars' trajectories based on individual scholars' lists of consecutive periodical publications.

**Figure 1:**
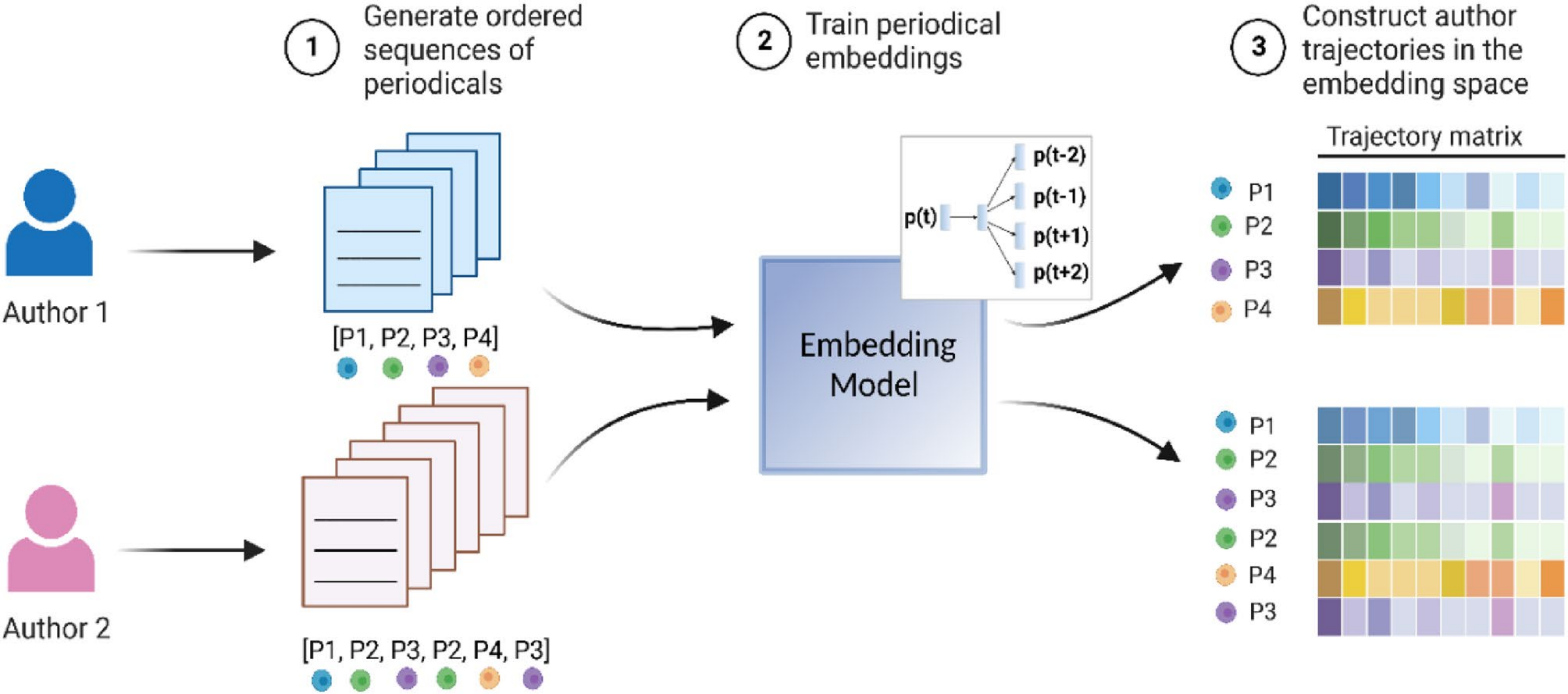
Construction of author trajectory matrix based on periodical embeddings. Starting from the author publication record—for example, Author 1 published four papers in four periodicals (i.e., P1, P2, P3, and P4), and Author 2 published six papers in the same four periodicals—step 1 generates ordered sequences of periodicals. In step 2, we train the periodical embeddings using the *word2vec*^20,21^ skip-gram implementation (i.e., to predict the context for a given periodical, as shown in the upper box). In step 3, we construct a trajectory matrix for each author based on the trained embeddings for each periodical. The rows represent the periodicals, and the columns represent the dimension of the embedding. The different colors in the matrix indicate different values.

As shown in Fig. 1, we use an embedding model to reconstruct the trajectory matrices. Specifically, we assemble and process a large corpus of 29,107 researchers who have used social media-derived data sets, consisting of 973,163 papers published during 1960–2019 in 26,815 periodicals, resulting in a total of 1,223,158 author-periodical combinations. This particular set of authors has diverse origins across multiple disciplines—spanning all main areas of science—while sharing contemporary interests in the data sets from social media sites, thus forming an interesting case study. While our dataset has an evident limit, we have a large number of publication trajectories to analyze, thus being able to rely on our methods and results more generally. We then apply the skip-gram algorithm^20–22^, which aims to predict context periodicals that appear in the proximity of the target periodical to learn the 100-dimensional embedding of the target periodical. After training, each periodical has a location in a geometric space, where periodicals that frequently share contexts are located nearby. More details about our dataset and model are included in the Material and methods section.

We discover that, even though no prior information or interpretation is provided to the model, the obtained periodical embeddings offer a powerful framework for quantitative examinations of individual and population trajectory patterns. We show that our novel approach can (i) monitor the author's publishing activity according to the locations of periodicals in a meaningful space, (ii) characterize the moving patterns based on statistical tools from mobility analysis, i.e., the radius of gyration^23^, and (iii) unravel the correlations between moving patterns in the periodical space and academic success.

## Results

### The space of scientific periodicals

Based on the trajectory corpus, we obtain a 100-dimensional embedding for each of the 11,909 periodicals (periodicals with less than ten publications in our corpus were dropped; see Material and methods section). Our embeddings encode the associations between periodicals, which can be represented as the geometric relationships between their embeddings in the vector space. We can measure periodical similarities via the cosine similarity between their embeddings^4^. When the cosine similarity between two periodical embeddings is high, one can expect that these two periodicals often appear together with similar context periodicals in the trajectory corpus. We observe that, for example, the most similar periodicals to *Proceedings of the National Academy of Sciences (PNAS)* are *Science* and *Nature*. The closest periodicals *to The International AAAI Conference on Web and Social Media (ICWSM)* are *The International ACM Conference on Web Science* and *arXiv: Social and Information Networks* (see Supplementary Information Table S1 for more details). This suggests that the positions of periodicals in the space capture subtle nuances of how periodicals can be substituted, such as the scope, field of study, infrastructure, and prestige.

**Figure 2:**
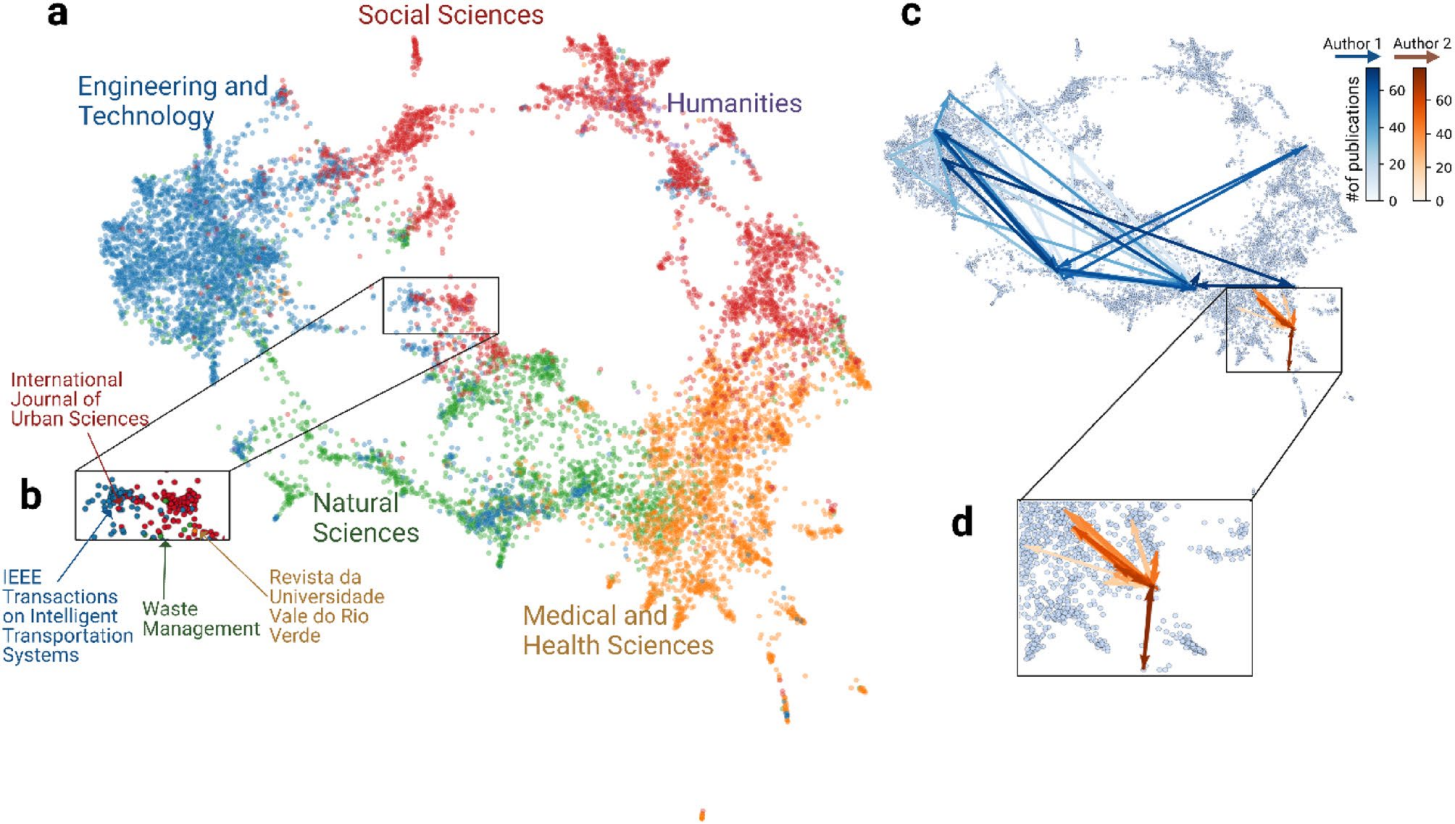
**(a)** The two-dimensional (2D) projection of 11,909 periodicals using the Pairwise Controlled Manifold Approximation Projection (PaCMAP)^24^. Each circle represents a periodical, and its color denotes its field category (an interactive version can be found at https://yangliu1231.github.io/periodical_embeddings). **(b)** A group of (inter-)disciplinary periodicals. For example, the *International Journal of Urban Sciences,* classified as "Social Sciences", and *IEEE Transactions on Intelligent Transportation Systems,* classified as "Engineering and Technology", are close together and located at the intersection of "Social Sciences", "Engineering and Technology", and "Natural Sciences". **(c,d)** Examples of author trajectory in the 2-d space. Here the blue arrow represents an author from "Engineering and Technology", and the orange arrow represents another author from "Medical and Health Sciences". The lightness of the color indicates the publication sequence. We can see that these two authors with comparable publication counts tend to have different movements in the periodical space.

We find that the obtained embeddings also capture the large-scale structures in the periodicals, i.e., the complex (inter-)disciplinary structure of science. Figure 2a shows a two-dimensional projection of periodicals using the Pairwise Controlled Manifold Approximation Projection (PaCMAP)^24^, providing an overview of our embedding space along with five scientific fields. Here, we color each periodical according to its field category. The four main fields, i.e., "Social Sciences," "Engineering and Technology," "Natural Sciences," and "Medical and Health Sciences," clustered in distinctive regions on our projection map, and the more interdisciplinary periodicals appear on the borders between different fields (Fig. 2b). While various dimensionality reduction techniques could be used to transform and visualize the embedding space (see Supplementary Information Fig. S4 for visualizations using different projection techniques), we find that the PaCMAP projection demonstrates slightly better performance in capturing the global disciplinary structure. In particular, we systematically compare the pre-defined field categories and the *k*-means clusters obtained from the periodical embeddings using the element-centric similarity test ^4,25,26^. Interestingly, we find that the two classifications (i.e., pre-defined field categories and the clusters obtained from the periodical embeddings) are broadly similar except for the regions surrounding the interdisciplinary periodicals, such as *Australian Educational Computing*, *Biological Cybernetics*, and *Physics in Medicine & Biology* (see Supplementary Information Fig. S5 for details). Additionally, we compared the average distances between journals within the same *subfields* and *fields*. We find that our periodical embeddings successfully underscore the similarities between journals within the same *subfields* (see Supplementary Information Fig. S6 for details).

It is worth noting that our embedding model takes the trajectory as input and treats the periodicals as entities, with no prior interpretation added; instead, the knowledge is captured through the position of the periodicals in the trajectories, more explicitly, the context periodicals for the targeting periodicals. These results suggest that our periodical embeddings constitute a meaningful space that captures the nuanced relationships between local periodicals and complex (inter-)disciplinary structures globally^4^.

### The trajectory patterns

Next, we explore the trajectory patterns in this knowledge space. Figure 2c–d plots some examples of author mobility in the embedding space—as a researcher's career progresses, her publishing venue moves between different periodicals (i.e., journals and proceedings), forming a unique publication trajectory in the space of scientific periodicals. Prior studies have demonstrated that the embedding models can learn a systematic representation of mobility in the cases of institution locations^16^ and research topics^15^. Hence, we expect the reconstructed trajectories from the embeddings of scientific periodicals to provide meaningful information about individual scientific activities and career profiles.

Suppose scholars' trajectories in the embedding space are indeed capable of capturing career information. In that case, it is reasonable to expect that the trajectory patterns are associated with academic success. To explore such relations, we measure the mobility patterns from the trajectories. These trajectories have high-dimensional features (i.e., 100-*d* periodical embedding) and unequal lengths among scholars (i.e., the number of publications). To reduce the complexity of trajectory data, we use single quantitative measures. Here, we introduce four measures, including *the mean embedding distance*^16^, *the average distance to the midpoint, the radius of gyration in the original space, and the radius of gyration in the 2-d space*^23^. In particular, the radius of gyration, ${r}_{g}$, is a well-known indicator in mobility analysis that measures the characteristic distance traveled by an individual^27–30^. We use it to measure the trajectory volume, i.e., the spatial spread of the periodicals traveled by scholars from the trajectories' center of mass.

As for the external indicator of academic success, we use the *average citations per paper*, i.e., the sum of the citations received for periodical papers in the space divided by the number of papers. This indicator has been widely used in the literature^31,32^ to measure the average impact of an individual's research output. It does not increase with the number of papers and allows a comparison of scholars of different ages^33^. The average citations, however, tend to penalize high productivity and may fluctuate significantly because of extreme values^33,34^. Hence, we also use the *75*^*th*^* percentile citations*, i.e., the number below which 75% of the citations fall, as an alternative measure of citation impact. Prior research^34^ has shown that the high decile region of citations can characterize a given citation distribution and discriminate scholars in a statistically reliable way. See Supplementary Information Fig. S3 for details about the citation distribution.

**Figure 3:**
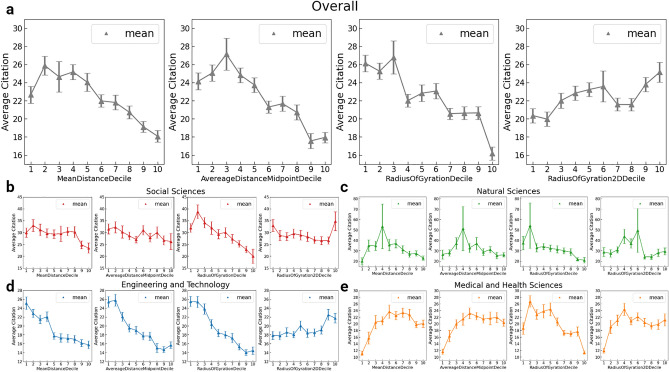
**(a)** The average citations for authors in the deciles of four trajectory measures: mean embedding distance, the average distance to the midpoint, the radius of gyration in the original 100-*d* space, and the radius of gyration in the 2-*d* space. **(b–e)** The average citations in the deciles for authors from different fields. Here we split the authors into ten equal-sized groups according to the deciles of the trajectory measures. For each group, we plot the mean with standard error bars.

We then investigate the relations between the trajectory measures and the academic success indicators. As shown in Fig. 3a, we split the scholars into ten equal-sized groups (i.e., deciles) according to the values of the trajectory measures. For each decile, we show the mean of the average citations, $\overline{c}$. In the case of *the mean embedding distance*, average citations rise to the peak in the second decile and gradually decrease. Similarly, the values in the deciles of *the average distance to the midpoint* steadily increase, peaking in the 3^rd^ group and gradually decreasing. Interestingly, this figure shows a rapid decrease in average citations in the deciles of the ${r}_{g}$ *in the original space* but a general increase in the deciles of the ${r}_{g}$ *in the 2-d space*.

Figure 3b–e further display the patterns for authors from different fields. For social scientists shown in Fig. 3b, we find that the ${r}_{g}$ groups demonstrate a relatively steady decline in average citations. No such clear patterns are found in the deciles of the other three measures. In the case of natural scientists (see Fig. 3c), what stands out is the high standard error in one of the decile groups, indicating a wide spread around the mean in that group. This high variance could be partly attributed to the large range in average citations as a result of the higher number of citations per paper in natural sciences than in other fields^35^. Nevertheless, we find the ${r}_{g}$ better indicates a trend in academic success across the decile groups than other measures, showing a marked decrease in average citations. For scholars from the field of "Engineering and Technology" (see Fig. 3d), all four measures demonstrate a clear trend of either increase or decrease across the decile groups. In the case of medical and health scientists (see Fig. 3e), we observe more fluctuations across these decile groups, in which the ${r}_{g}$ deciles show a relatively clear decreasing trend. We also observe the heterogeneous patterns across fields; for example, average citations in the deciles of *the average distance to the midpoint* show a general decreasing trend but an increasing trend in the case of "Medical and Health Sciences".

Overall, we find that, unlike the fluctuations observed in other trajectory measures, the patterns of the ${r}_{g}$ *in the original space* are generally consistent across different fields, highlighting a steady decline in academic success when the ${r}_{g}$ increases. Our results suggest that staying relatively still in the embedding space of periodicals through publishing consistently (i.e., exploitation) would generally be associated with a higher citation success than extensive changes in publishing venues (i.e., exploration) or a combination of both strategies (i.e., a combination of exploration and exploitation). Similarly, we find that the ${r}_{g}$ *in the original space* demonstrates the best performance to discriminate the academic success between different decile groups when using the *75*^*th*^* percentile citations* as the success indicator (see Supplementary Information Fig. S7 for details). Consequently, we consider the radius of gyration a reliable measure of a scholar's trajectory in the space of scientific periodicals.

**Figure 4:**
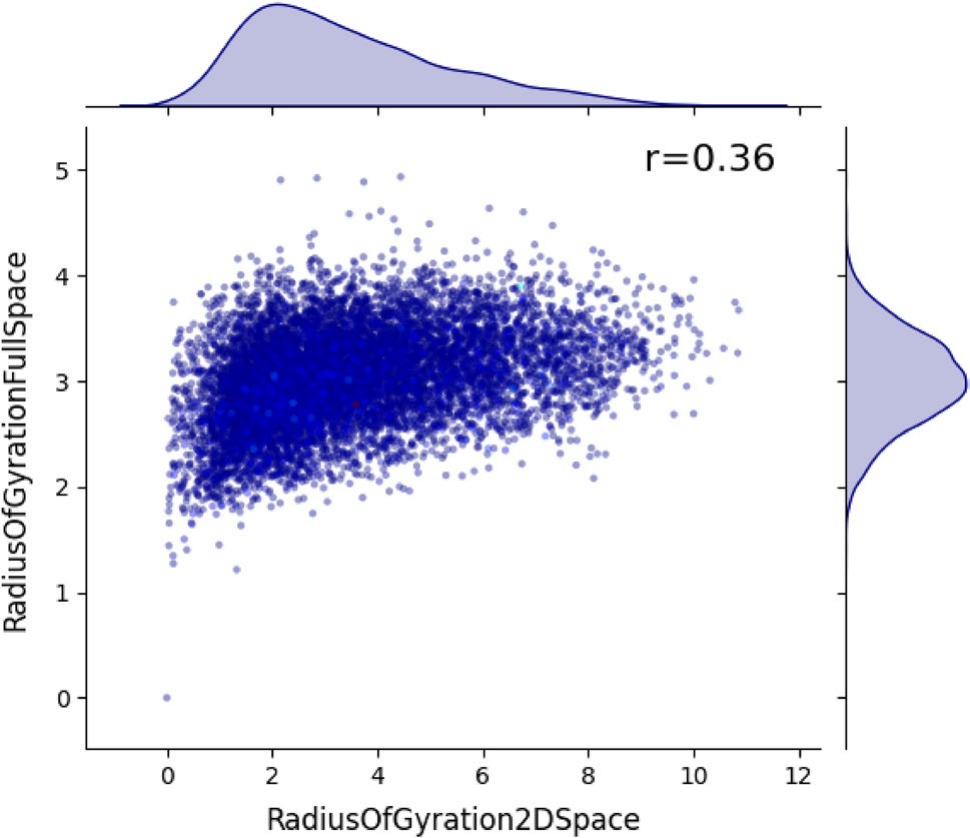
The radius of gyration in the 2-*d* space vs. the radius of gyration in the original space. Here each point indicates a scholar, and the point's color in a gradient from blue to red indicates the average citations. We also show probability density curves for both measures. We can see that these two measures are roughly correlated, with r showing the Pearson correlation coefficient ($p<0.001$).

To explore the striking differences between ${r}_{g}$ in the 2-*d* and original spaces, we show the relations between these two measures for the same individuals (See Fig. 4). We find a nontrivial positive correlation with Pearson correlation coefficient ${\text{r}}=0.36, p<0.001$. Interestingly, this figure reveals that ${r}_{g}$ in the original space tends to have a more restrictive range that it is hard to achieve low or high values. In other words, most scholars would have relatively more comparable mobility patterns in the original space, measured through the trajectory volume. While examining the correlation between average citations and ${r}_{g}$, we observe a notable shift in trends between the 100-dimensional space (negative correlation, r = -0.089, p < 0.0001) and the two-dimensional PaCMAP space (positive correlation, r = 0.031, p < 0.01). This suggests that the PaCMAP transformation highlights certain structures or features that are less dominant in the original high-dimensional space. Due to the nonlinear nature of PaCMAP, the 100-*d* and 2-*d* spaces are not directly comparable. We therefore use the Principal Component Analysis (PCA)^36^ as a linear baseline to explore the changes in the space when we gradually reduce the dimensions. We find that the correlation trend also switches with the linear dimensionality reduction, reflecting the loss of information inherent in reducing dimensions of periodical embeddings (see Supplementary Information Fig. S8).

A manual investigation shows that scholars with low ${r}_{g}$ values in both spaces rarely move between different venues, i.e., they tend to publish their work in the same periodicals. In contrast, scholars with high values in both spaces tend to publish in diverse periodicals. When looking into the authors with high values in the original space while low values in the reduced space, we find that they tend to publish in different periodicals that are closed together in the 2-*d* space. While both embedding spaces encode the relationships between periodicals, there are still many differences. As previously discussed, we believe that the 2-*d* space of scientific periodicals is primarily about the (inter-)disciplinary structure (see Fig. 2a). The original 100-*d* space thus captures many other directions that the periodicals can differ. Taken together, these results show that the ${r}_{g}$ in the original space is a reliable indicator that captures the more nuanced mobility patterns of scholars' trajectories.

### Regression models

We perform regression analyses to learn more about the relationship between mobility in the embedding space and academic success. We use the ${r}_{g}$ in the original space as an independent variable and the citation indicators as dependent variables. Empirically, when observing the dynamics in the radius of gyration as a function of publication number, we find that ${r}_{g}$ demonstrates a relatively stable behavior when the publication number is over twenty (See Figs. S9 and S10). Hence, we restrict our dataset to authors with more than twenty publications in the embedding space. Prior studies demonstrate the differences in received citations across gender, disciplines, countries, and age ^35,37–42^. It is thus desirable to control for these variables. We estimate the coefficients using the ordinary least square method (OLS). We have checked the absence of multicollinearity and used unbiased standard errors of the coefficients.

Table 1 presents the model regressing average citations on ${r}_{g}$ in the original space. We observe that author mobility in the periodical embedding space is negatively associated with average citations: a one standard deviation increase in the radius of gyration is associated with a decrease in average citations by 5.28, when controlling for the author's gender, academic age, region, number of publications, and disciplinary background (see Supplementary Information Table S2 for a full table showing the coefficients of each variable). In addition, the heterogeneity analysis reveals a statistically significant negative relationship between ${r}_{g}$ and academic success for authors of different gender, disciplines, regions, and age groups. The results remain similar when we measure the log-transformed citations and the 75th percentiles of citations (see Supplementary Information Table S2 for details). Hence, the regression analyses reveal that the author's mobility measured through the ${r}_{g}$ in the original space exhibits a significantly negative correlation with the academic success indicators.

**Table 1:** The linear regression model for academic success.

	Average citations
The standardized radius of gyration	−5.28*** (0.39)
Control variables (gender, academic age, region, number of publications, disciplinary background)	Yes
R-squared	0.13
R-squared adj	0.13
N	10,480

Robust standard errors in parentheses.

$$*p< 0.05, **p<0.01, ***p<0.001$$.

Additionally, we regressed ${r}_{g}$ in the 100-*d* space on PaCMAP 2-*d* ${r}_{g}$, isolating the portion of ${r}_{g}$ in the 100-*d* space that is not explained by the 2-*d* space. The residuals from this analysis have a negative correlation with citation indicators. As previously mentioned, we conceptualize the 2-*d* space as primarily representing the (inter-)disciplinary structures in science. This implies that, in a broad sense, non-disciplinary movements or factors not related to the primary disciplinary structure are *negatively* correlated with citation success.

## Discussion and conclusions

We present an embedding-based framework as a first attempt to (i) track scholars' trajectories across periodicals, (ii) use the radius of gyration to capture the complex trajectory patterns among scholars in the periodical space, (iii) associate the scholars' academic success with their trajectories in the space of scientific periodicals.

We reconstruct 29,107 social media researchers' trajectories containing 973,163 papers published between 1960 and 2019 in 26,815 periodicals. While there is substantial literature on reviews of social media research^43–50^, this study contributes to our understanding of this fast-moving, interdisciplinary field by providing the publishing histories of individuals who have conducted social media research. We expect the periodical embeddings trained on this large-scale trajectory dataset to provide new ways to scrutinize scholars' scientific activities.

We conceptualize periodicals—referring to both journals and proceedings—as highly granular subdivisions of science, encompassing both field and status distinctions. We use an embedding model to capture the complexities of how periodicals can be functionally substituted for one another, based on the dynamics and nuances in how authors traverse the space of periodicals. The trained periodical embeddings form a meaningful knowledge space that captures the subtle nuances between periodicals at the local level and complex (inter-)disciplinary structures at the global level. This space further provides a powerful framework for quantitative examinations of trajectory patterns by allowing geometric algebra operation among periodicals.

To characterize the trajectory patterns, we extend the human mobility measure, the radius of gyration, from the geographic realm to the geometric realm^15,23^. Many other measures can be studied concerning individual trajectory patterns, such as the embedding-based distance^16^, the Shannon Entropy^51^, and the convex hull area^52^(See Fig. S12 for the correlation between an example set of measures). Empirically, we find the radius of gyration to effectively discriminate authors regarding their academic success compared to other mobility measures*.*

We translate these trajectory patterns into academic success, controlled by author-based variables such as gender, regions, disciplinary backgrounds, and academic ages. We find that mobility in the periodical embedding space is negatively correlated with citation impact. In other words, the more successful scholars in the studied sample generally tend to publish in a relatively proximal range of periodicals. This negative association holds for (social media) researchers in different disciplines, regions, genders, and career lengths.

Our result, to some extent, corresponds to previous studies on the relations between academic success and interdisciplinarity, as well as the trade-offs between exploration and exploitation^19,53,54^. Research on exploitation (of established certainties) and exploration (of new possibilities) suggests that the returns from exploitation are systematically more certain compared to exploration^53^. We find that adopting an exploitation strategy, particularly through consistent publishing in a closed range of periodicals, is linked to greater academic success than exploration or a combination of both strategies (i.e., exploration and exploitation). Moreover, earlier studies demonstrate that academic success is best achieved by focusing on a narrow research area or related fields of knowledge^15,54^; the interpretation is that researchers tend to have bounded rationality and cognitive proximity such that the distant explorations in the knowledge space are associated with uncertainty and may lead to failure in terms of citation impact. Additionally, previous research argues that the highly innovative, unconventional knowledge combination may be challenging for scientific audiences and thus harm the citation impact. In our space of scientific periodicals, while the trajectory reflects the scholar's exploration of the knowledge space, it also captures the movements across other aspects of periodicals, such as the prestige, infrastructure, readership, performance, and peer review, corresponding to many underlying factors of journal selection criteria^6–9^.

On the one hand, our findings align with the widely-held view that academia generally gives less credit to highly innovative research that conjoins distant bodies of knowledge. The second interpretation of our findings is that scholars who publish in a vast volume of periodicals may signal the inability to have a regular audience and thus fails to achieve an increased reputation as their work is exposed to a constantly changing readership^54^. Another potential reason is that these highly-mobile scholars may experience more rejections that force them to change their publication venues and result in a large trajectory volume in the space of scientific periodicals.

We note several limitations of our study that deserves future research efforts. First, our periodical embeddings may reflect limitations in the data. Our dataset is limited to a particular set of scholars who have used social media data. While we filter the periodicals with less than ten papers, the effective samples for each periodical may be limited (See Fig. S2 for the number of papers per periodical). The periodicals with fewer papers may have less accurate embeddings. Future studies can extend our study in data collection to achieve a broader sample of scholarly publications. Second, the current analysis does not reveal the context and mechanism of trajectory patterns. Future research might extend our framework to investigate the collaborative patterns, author positions, and roles, as well as fundamental principles governing scholars moving between periodicals. Third, our study has the known limitations of the neural embedding approach, such as unknown biases and interpretability problems^4^. Another important limitation pertains to the application of human mobility measures (e.g., radius of gyration) to the high-dimensional space, in which each dimension contributes equally to the measure. Even though we carefully validated our approach, there may be unknown biases in such applications, as the exact properties of the dimensions in the periodical embeddings have not been fully understood. Future studies may examine biases in the embeddings of scientific periodicals, such as whether the embeddings for multidisciplinary periodicals are skewed, and attempt to determine the optimal embedding dimensionality for mobility analysis. Other data types (e.g., paper or periodical citations) and the more advanced models may also be used to train periodical embeddings. A dynamical embedding model, for instance, could reveal the potential drifts of periodicals in the space over time. Fourth, our study only uses citations to measure academic success. Future work can test our results using other measures, such as cognitive innovation and faculty positions. Fifth, the Gender API was unable to infer genders for authors lacking full first names in the dataset or authors based in certain regions, particularly in East Asia (e.g., China, Japan, and South Korea). Finally, we should stress that our analysis does not derive any causal relations because there may be certain confounders that are not observed or controlled for, or because of reverse causation that less successful researchers may have to try different periodicals and hence have higher ${r}_{g}$. Besides, the current regression estimates only describe the average effect and assume a linear relationship between the trajectory patterns and scientific success. Therefore, it does not reveal any potential dynamics or nonlinear relationships. Notwithstanding these limitations and uncertainties, we believe our methods and findings have important implications for the literature on science studies and mobility analysis beyond the geographic realm more generally.

## Material and methods

### 1. Dataset

We use an original dataset to explore the trajectory patterns of authors, consisting of 973,163 publications from 1960 to 2019 for 29,107 authors selected from a sample of researchers who have used social media data. We focus on these researchers because social media data sets accommodate research interests and expertise from a wide range of disciplines, from *media studies* to *computer science*, as well as emerging interdisciplinary research fields such as *computational social science* and *digital humanities*. This particular group of scholars, while being largely disconnected and fragmented, have shared attention and thus formed an interestingly diverse case to explore scholars' trajectories more broadly. We first query a set of social media data-based research from the Web of Science (WOS) in SCI-EXPANDED, SSCI, AHCI, and ESCI, between 2005 and 2019. The search strings are (TS = ("social media data")) OR (TS = ("social media" OR "social network* site*" OR "online social network*" OR facebook OR twitter OR instagram OR blog OR microblog OR YouTube OR Flickr) AND TS = (tweet* OR hashtag* OR weblink* OR posting OR commenting OR "news feed" OR user profile OR geotag* OR Youtube video OR "digital data" OR "computational social" OR "digital social" or "social web data") NOT TS = (survey OR questionnaire OR interview)) OR (TS = ( facebook OR twitter OR instagram OR blog OR microblog OR YouTube OR Flickr) AND TS = (dataset)). We check the quality of our dataset and confirm that this dataset is a valid and conservative approximation of research using social media data.

Based on the queried 12,732 research papers, we source publication trajectories for relevant authors from the Microsoft Academic Graph (MAG) dataset^55,56^, capturing 29,107 authors. Author publication trajectories are constructed based on publication venues listed in MAG, including journals and conferences. Given the name disambiguation issue of MAG data^57^, we limit our dataset to papers published after 1960. This yields 1,223,158 author-periodical combinations, representing 973,163 papers published in 26,815 periodicals by 29,107 authors between 1960 and 2019 (See Figs. S1 and S2). Each author-periodical record is linked to a MAG author ID, a MAG venue ID, and the published date and year. Trajectories are generated as the sequence of author-periodical records for each author, ordered by the date of publication.

Besides, we collect all available information for individual authors in the MAG dataset, including affiliations, MAG Field of Study (MF), and received citations from each publication. We use the author's last known affiliation to determine the geolocation. We calculate the gap between the year of the first publication and 2022 as their academic ages. For the assignment of disciplinary background, we use the majority of top-level MFs of the author's publications. Furthermore, we assign gender to each author's first name based on the prediction results of a commercially available service, *Genderize.io*, which has been employed in the literature focusing specifically on gender inequality in science^58–60^. Due to methodological and data limitations in name gender disambiguation, we are unable to assign genders to 28% of authors (labeled as "unknown"), systematically lacking the gender information for authors in specific regions, particularly in East Asia (See SI, section S1 Gender assignment). We also estimate the fields of periodicals based on most top-level MFs of the publications. Note that we map the 19 top-level MFs into five broad field categories—"Social Sciences", "Engineering and Technology", "Natural Sciences", "Humanities", and "Medical and Health Sciences"—based on the OECD Field Category^61^.

### 2. Embedding model

To measure the statistical properties of the trajectory patterns in a geometric space, we embed author publication sequences using the *word2vec* model^21^, by treating a periodical as an entity and an individual publication trajectory as a sequence (see Fig. 1). Instead of using the paper citation network of the periodicals^4^, our model leverages the more explicit publishing sequences of author trajectories to learn the nuanced relationships between periodicals based on how authors journey through science. We use the Skip-gram algorithm to find periodical representations that effectively predict the context periodicals in a publication trajectory. The objective function is to maximize the average log probability^17^,

1 $$O=\frac{1}{T}\sum_{t=1}^{T}\sum_{-c\le j\le c,j\ne 0}{\text{log}}p\left({p}_{t+j}|{p}_{t}\right)$$

where *p* is the center periodical, *c* is the size of context periodicals, and *T* is a sequence of periodical publications (${p}_{1}, {p}_{2}, {p}_{3}, \dots , {p}_{T})$.

We use the Word2vec skip-gram implementation in the Python package genism (https://radimrehurek.com/gensim/models/word2vec.html) that takes trajectories as input, with the vector size of 100, min_count of 10, window size of 2. The window size of two is determined based on our inquiry's nature to capture the transition between two consecutive periodical publications. We tune other hyperparameters based on a qualitative examination of similarities between periodicals. After training, we obtained a vector representation for each of the 11,909 periodicals that occurred at least ten times. The distance between periodicals relates to their occurrences in the same contexts. We further repeated our analysis using a much lower number of dimensions. We found that the high-dimensional embedding space provides better vector representations of the periodicals regarding the nuanced discriminative information among periodicals, along with a more effective tool for tracking authors' publishing activities and characterizing different mobility patterns (See SI section S3 for an embedding model with five dimensions).

### 3. Measure definition

We introduce a set of measures of trajectory patterns, including *the mean embedding distance*, *the average distance to the midpoint, the radius of gyration in the original space*, and *the radius of gyration in the 2-d space.* We first estimate the embedding distance between the author's locations at consecutive publications, capturing 1,006,522 displacements in our trajectory data. Instead of calculating the total distance for each author, we use the mean distance to estimate the author's average movement. As such, we capture the characteristics of their movements while mitigating the effect of publication number. We calculate the distance between locations of periodicals *d*_*ij*_ based on the cosine similarity,

2 $${d}_{ij}= 1-\frac{{\overrightarrow{p}}_{i}\cdot {\overrightarrow{p}}_{j}}{\parallel {\overrightarrow{p}}_{i}\parallel \parallel {\overrightarrow{p}}_{j}\parallel }$$

where ${\overrightarrow{p}}_{i}$ and ${\overrightarrow{p}}_{j}$ are the embeddings for periodicals *i* and *j*, respectively. In a recent study of trajectory embeddings^16^, this cosine similarity-based embedding distance outperforms other forms of distance measures. Likewise, we estimate the average distance between individual publication positions and the center of the trajectory, $\overline{d }$, using this cosine similarity-based embedding distance,

3 $${\overline{d} }=\frac{1}{N}\sum_{i\in K}{{{n}_{i}d}_{i,cm}}$$

where *N* is the total number of publications, *K* is the set of periodicals visited by an individual, ${n}_{i}$ is the number of visits of the periodical *i*, cm is the trajectory's center of mass, i.e., the weighted mean of the periodicals visited by the author.

Besides, we also calculate the radius of gyration, *r*_*g*_, for each author, interpreted as the mobility volume^23^, characterizing the spatial spread of the positions visited by an individual from the center of mass of the trajectory,

4 $${r}_{g}=\sqrt{\frac{1}{N}\sum_{i\in K}{{n}_{i}({r}_{i}-{r}_{cm})}^{2}}$$

where *N* is the total number of visits, *K* is the set of periodicals visited by an individual author, ${n}_{i}$ is the number of visits of periodical *i*, ${r}_{i}$ is the vector of periodical *i*, and ${r}_{cm}$ is the center of mass.

### Supplementary Information

Supplementary Information.

## Data Availability

The dataset, embedding model, along with code for analysis, have been published at https://github.com/YangliuF95/Author_trajectory.
