# Supplementary material for: Understanding scholar-trajectories across scientific periodicals
Source: Sci Rep. 2024 Mar 4;14:5309. doi: 10.1038/s41598-024-54693-7 (PMC10912201; doi:10.1038/s41598-024-54693-7)
Supplement: Supplementary file 1 — Supplementary Information. [file 41598_2024_54693_MOESM1_ESM.pdf]

Supplementary Information for

Understanding scholar-trajectories across scientific periodicals

Yangliu Fan[1,*], Anders Blok[1,2], Sune Lehmann[1,3]

[1]Copenhagen Center for Social Data Science, University of Copenhagen, Copenhagen, Denmark.

[2]Department of Sociology, University of Copenhagen, Copenhagen, Denmark.

[3]DTU Compute, Technical University of Denmark, Lyngby, Denmark

*Correspondence: Yangliu Fan, yangliu.fan@weizenbaum-insitut.de

1. Gender assignment

1.1. Gender inference

Due to the absence of gender information in the MAG dataset, we inferred the author's gender based on their first names. We used a commercially available service, *Genderize.io* (https://genderize.io/), which has been widely employed in the literature, focusing specifically on gender inequality in science [1–3]. Although *Genderize.io* is shown to achieve relatively good gender assignment performance [4], we observed a low certainty score (<0.8) for a considerable number of authors in our dataset, i.e., 28% of authors are labeled "unknown." Close inspection reveals that more than half of these authors lack full first names in the dataset. Notably, 643 "unknown" authors are affiliated with East Asian institutions in South Korea, Japan, Mainland China, Hong Kong, Taiwan, and Macao. For instance, among 765 authors affiliated with institutions in Mainland China, *Genderize.io* could not infer the gender of 364 (47.6%) authors.

1.2. East Asian researchers

The reliability of name gender disambiguation for East Asian authors has been recognized as a common limitation in many existing gender studies. For instance, previous research excluded authors from certain countries, including China, the Democratic People's Republic of Korea, Japan, Malaysia, the Republic of Korea, and Singapore, due to the challenges in gender inference [1].

To assess the accuracy of gender assignment for East Asian researchers in our studied sample, we compared the inferred gender label ("male" or "female") based on *Genderize.io* with the gender information of Web of Science (WoS) authors in a dataset from Larivière et al. (2013). In their study, gender was obtained by matching names with universal and country-specific name lists. Specifically, we matched the *author's first names* and *publication DOIs* with their WoS dataset. Among the 216 East Asian researchers identified in their dataset, 198 have inferred gender information that aligns with their labels, resulting in an accuracy of 91.7% (if we consider their gender assignment as the ground truth).

To further investigate this potential bias, we conducted a manual check. Specifically, we randomly sampled 50 authors affiliated with East Asian institutions and manually collected their gender information. Our search used a combination of their full names, article DOIs, and institution names to identify the authors. We checked their official websites, pronouns, and profile pictures. As a result, we found 39 male researchers, nine female researchers, and two researchers for whom we were unable to find relevant gender information. Among the 50 authors, 25 have inferred gender labels from *Genderize.io* that agree with the ground truth, resulting in an accuracy of 50%. Among the 25 authors with misinferred gender information, the majority (23) are labeled "unknown" by *Genderize.io*. We are therefore confident that *Genderize.io* has demonstrated reliable performance for those labeled as "male" or "female" authors. Nevertheless, it has limited ability to infer genders for East Asian researchers.

2. Tables and figures

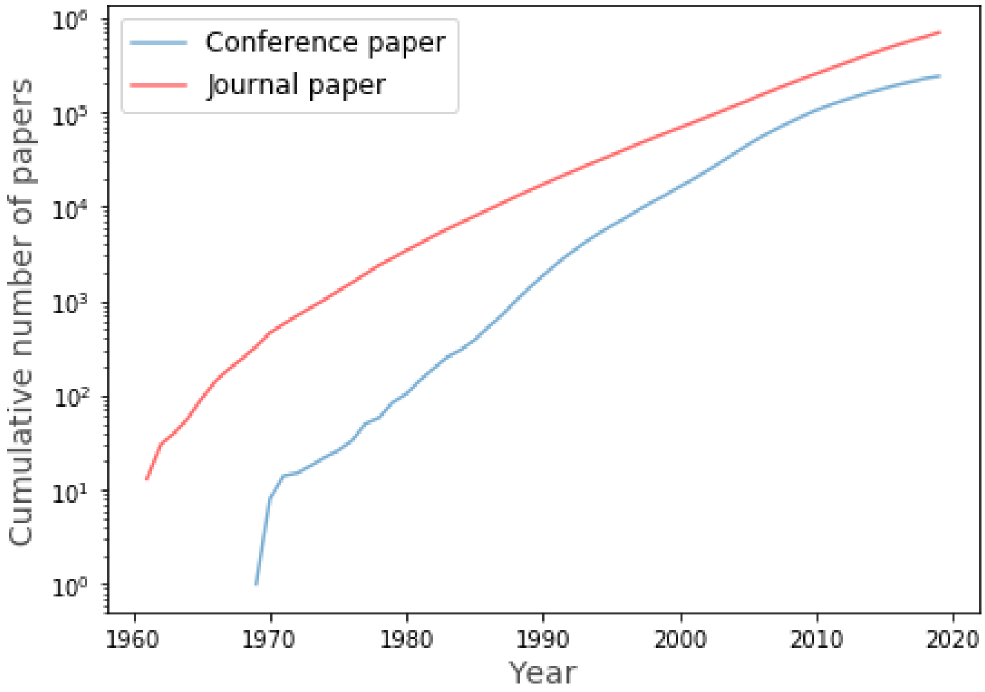

Figure S1. The total number of journal and conference papers from 1960 to 2019.

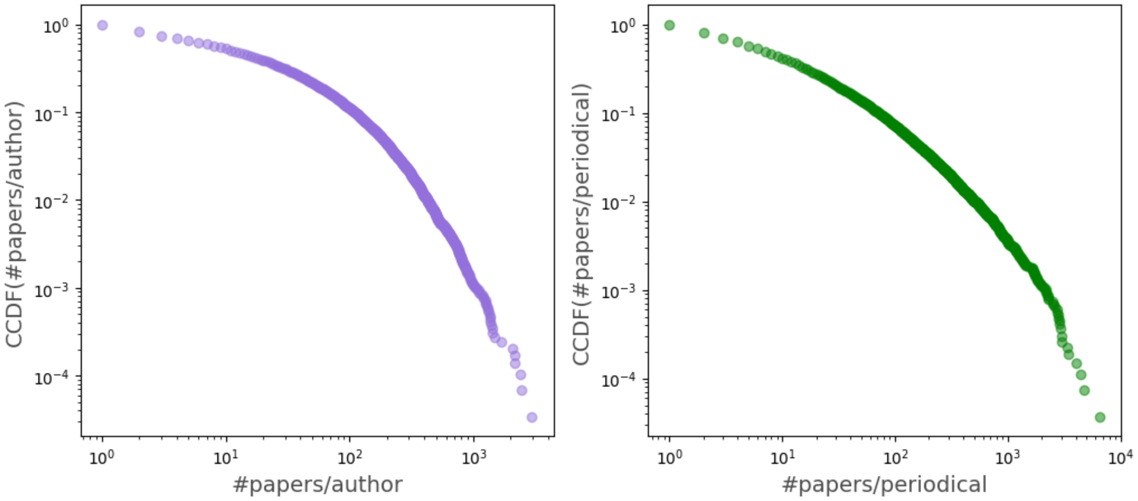

Figure S2. Complementary cumulative distribution function (CCDF) of the number of papers per author and the number of papers per periodical.

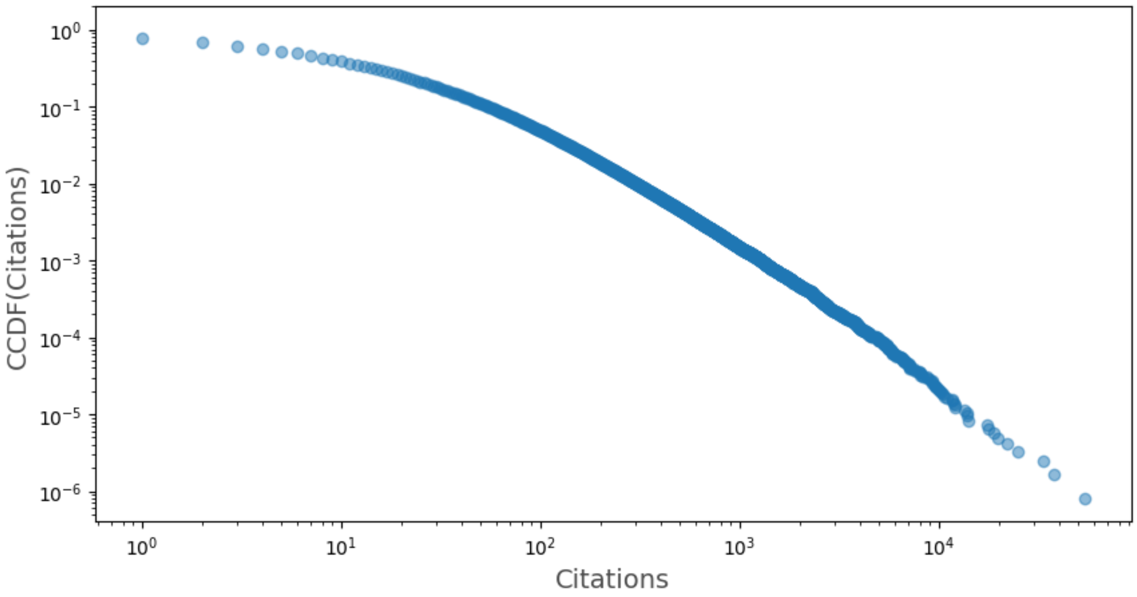

Figure S3. Complementary cumulative distribution function (CCDF) of citations per paper.

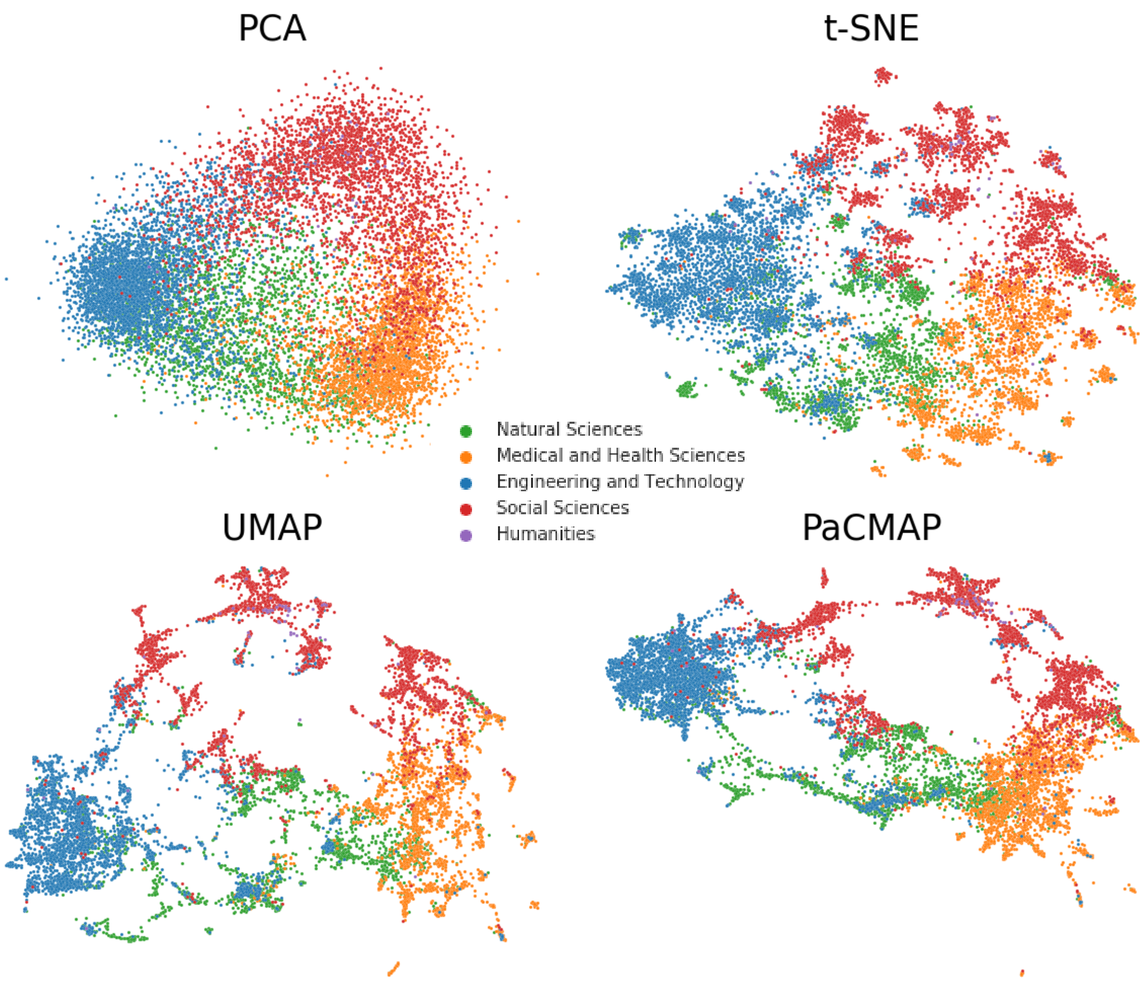

Figure S4. Comparison of the PCA, UMAP, t-SNE, and PaCMAP[6–9] visualizations for the high dimensional periodical embeddings, colored by field categories.

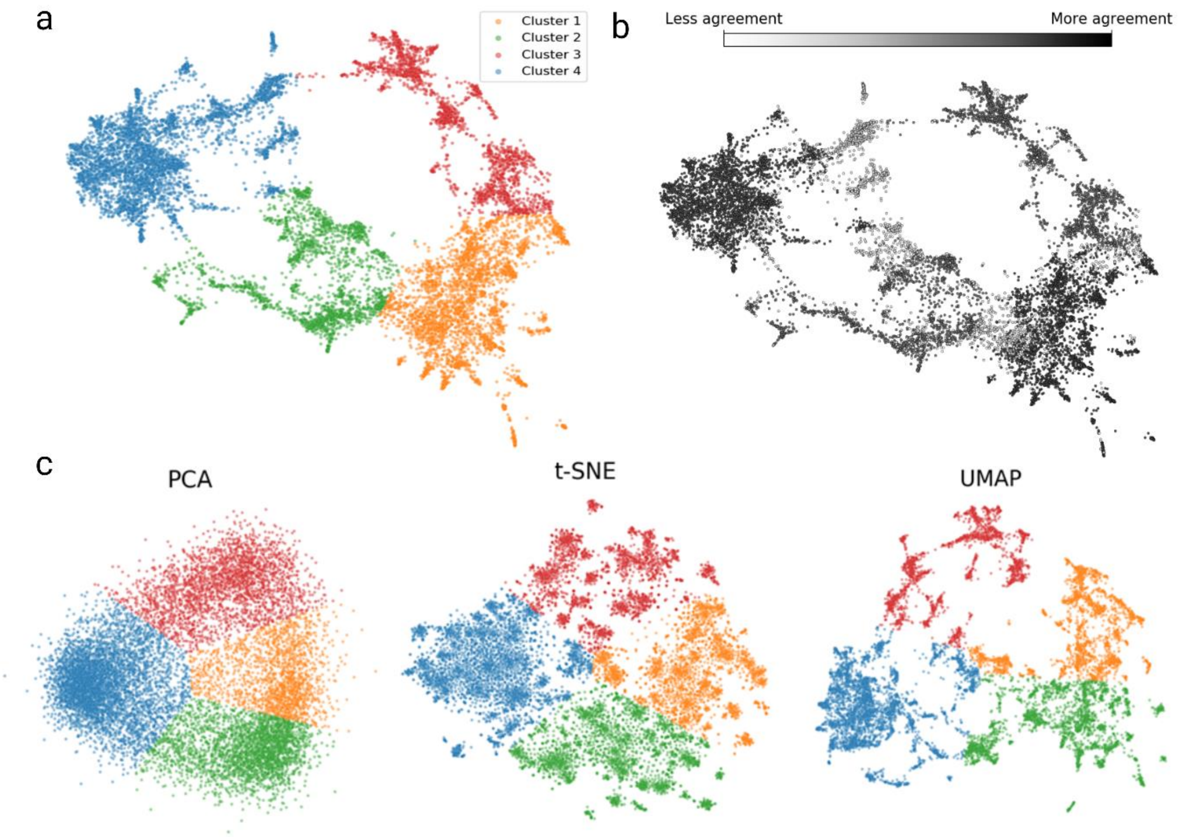

Figure S5. (a) The PaCMAP projection of the periodical embeddings, colored by the four *k*-means clusters[10] (b) The PaCMAP projection of the periodical embeddings with a grayscale representing the level of agreement between the four *k*-means clusters and the four field categories. Here, the level of agreement is obtained using the element-centric similarity test[11]. We find that the more disciplinary periodicals show higher agreements between the two clusterings, while interdisciplinary periodicals exhibit lower agreements. (c) The 2D projections of periodical embeddings, colored by the four *k*-means clusters. We find that the PaCMAP projection achieved a slightly better similarity score (0.558) than the PCA (0.498), t-SNE (0.492), and UMAP (0.483) projections.

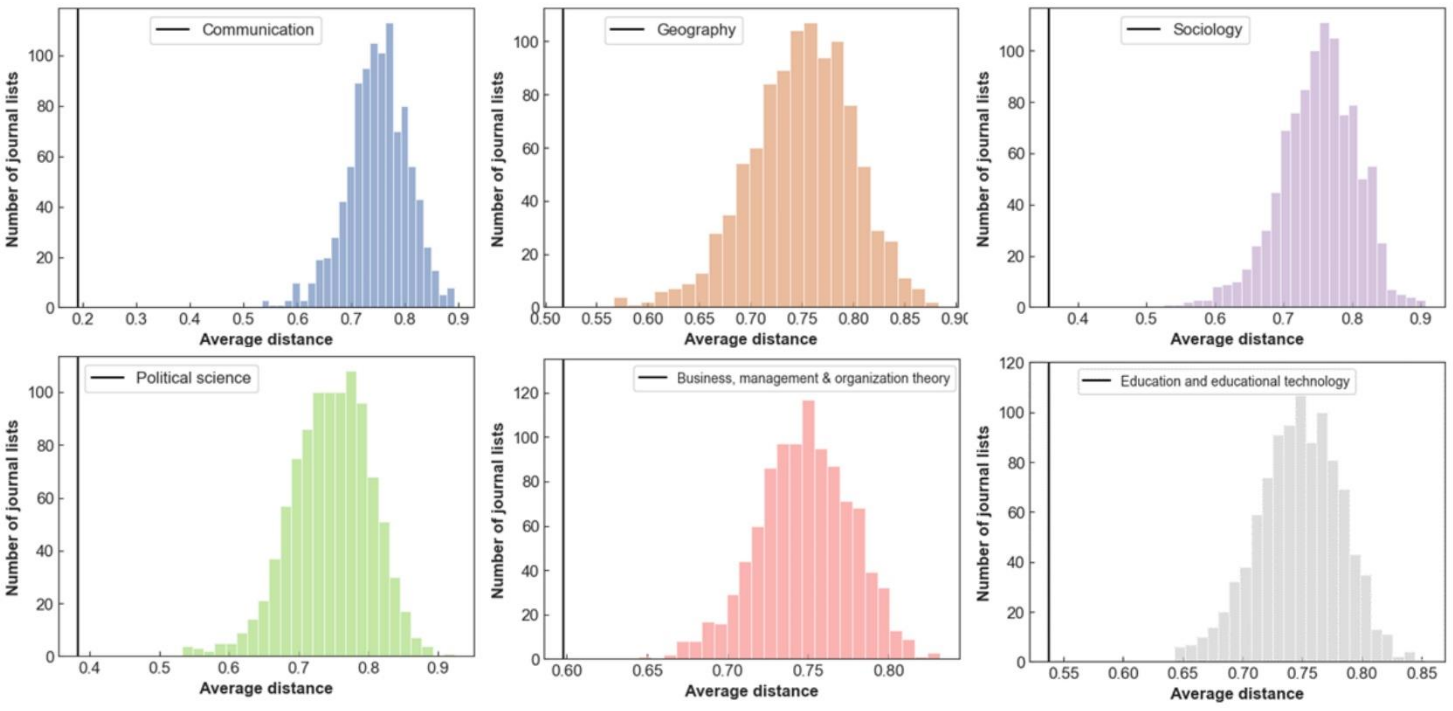

Figure S6. The average distance between journals within the same *subfield* (black line) and the distribution of the average distance between the same number of journals within the same *field*, based on 1000 randomly selected journal lists (color bars). We evaluated our embeddings based on a cognitive structure provided by Wikipedia, in which journals are organized into *fields* (e.g., social science) and *subfields* (e.g., communication, business, and education). Specifically, we matched our trained embeddings with the journals in the Wikipedia catalog (https://en.wikipedia.org/wiki/List_of_social_science_journals). We first calculated the average embedding-based distance between journals from the same *subfield*. We compared these *within-subfield* distances with 1000 instances of journal lists, each representing the average distance between the same number of journals randomly sampled from the catalog (i.e., *within-field* distance). The figures illustrate that the average distance within *subfields* is significantly lower than the null model (within *field*). Therefore, this test suggests that the periodical space underscores similarities among journals within the specific *subfields* in a defined cognitive structure.

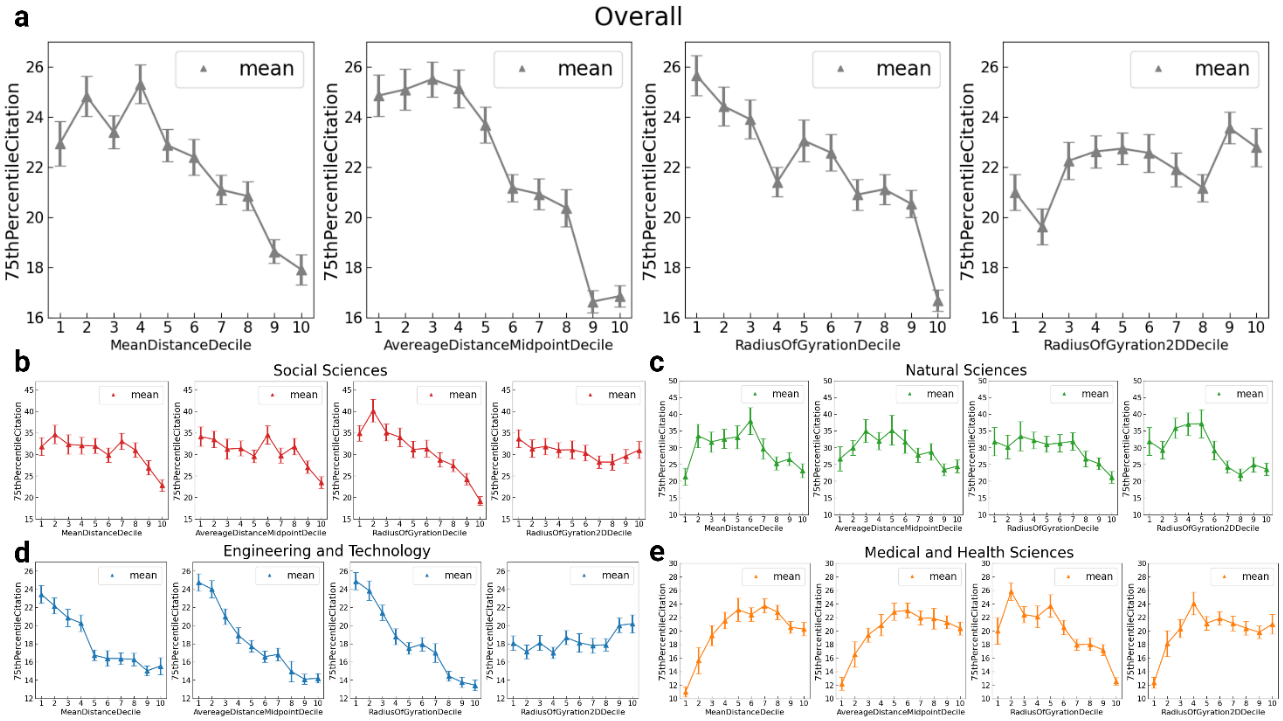

Figure S7. (a) The 75th percentile citations for authors in the deciles of four trajectory measures: mean embedding distance, the average distance to the midpoint, the radius of gyration in the original 100-*d* space, and the radius of gyration in the 2-*d* space. **(b-e)** The 75th percentile citations in the deciles for authors from different fields. Here we split the authors into ten equal-sized groups according to the deciles of the trajectory measures. For each group, we plot the mean with standard error bars.

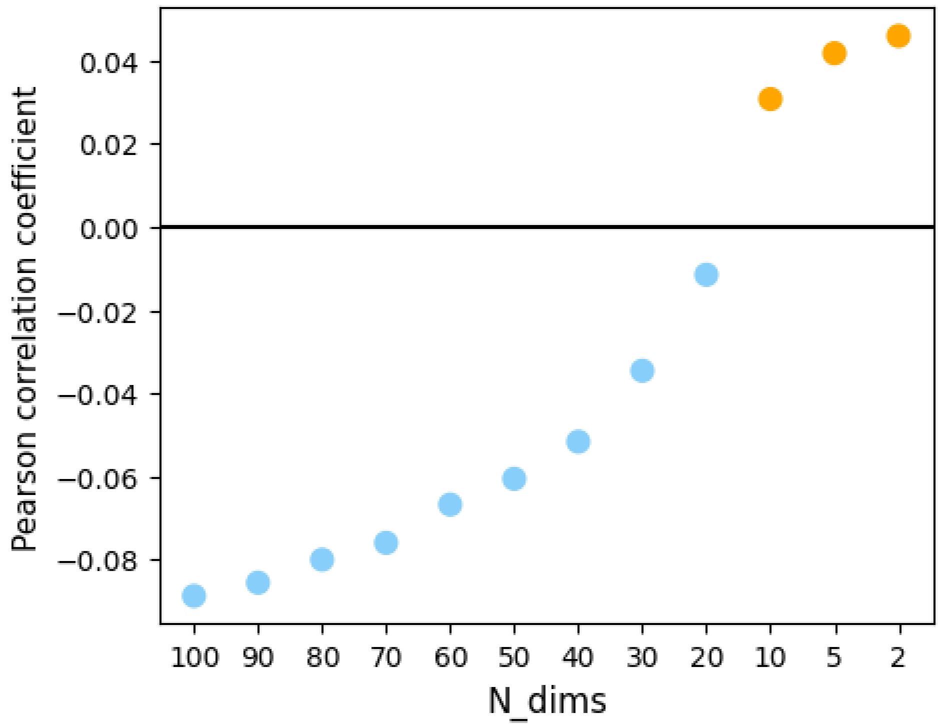

Figure S8. The Pearson correlation coefficients between the average citation and the radius of gyration. We use the PCA as a linear baseline to investigate the changes in coefficient. Specifically, we gradually reduced the dimensions from 100 to 2 dimensions using the PCA, retaining 90, 80, 70…10, 5, and 2 dimensions. We find that even with the linear transformation, the correlation switches as we move from higher to lower dimensions.

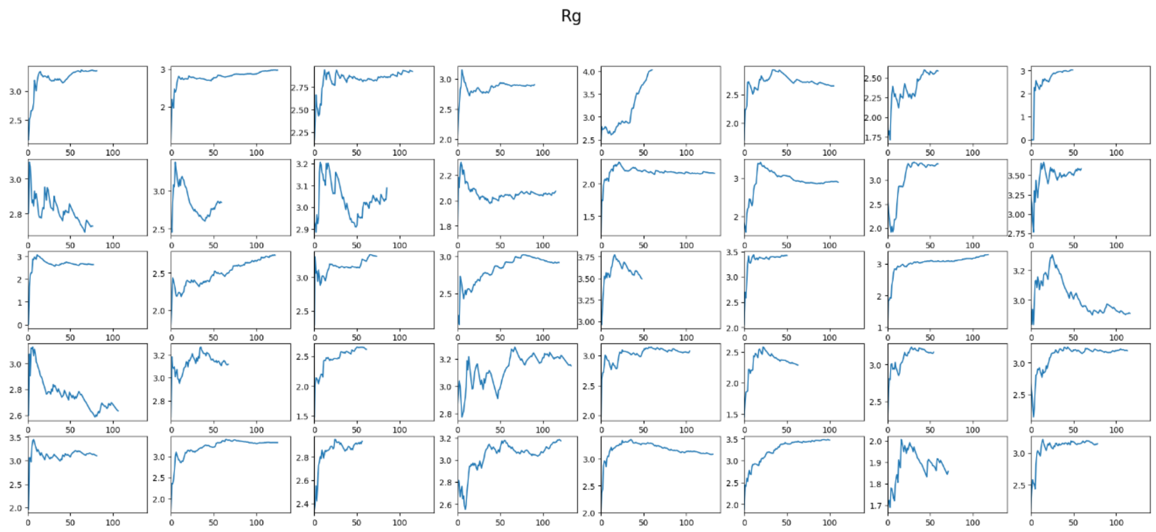

Figure S9. The radius of gyration (Rg) in the original 100-dimensional space, as a function of publication number for randomly sampled 40 authors. Here the x-axis shows the number of publications, and the y-axis shows the value of Rg.

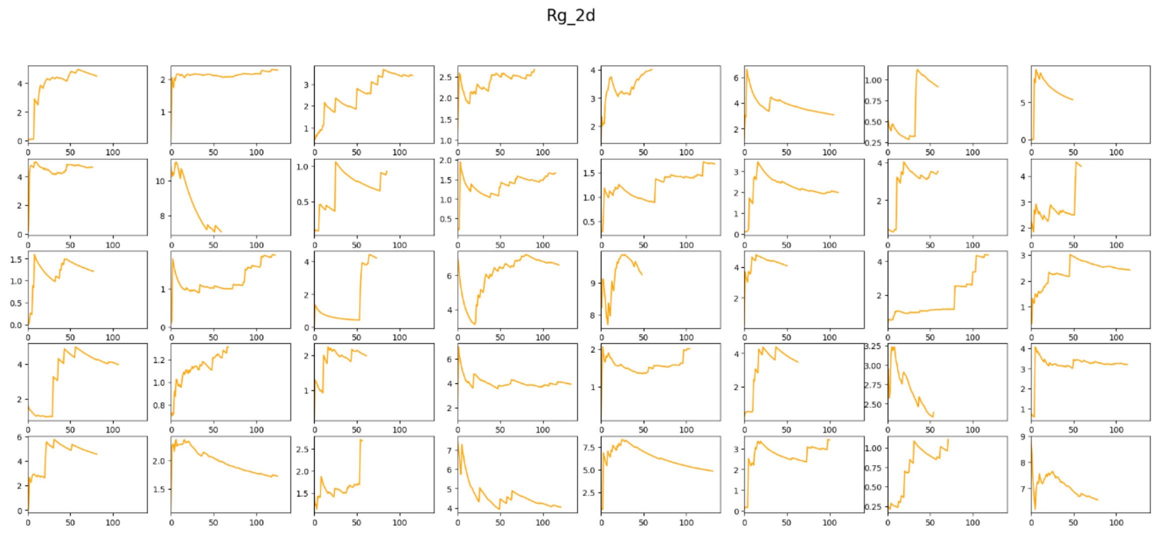

Figure S10. The radius of gyration (Rg) in the two-dimensional space using the PaCMAP algorithm, as a function of publication number for randomly sampled 40 authors. Here the x-axis shows the number of publications, and the y-axis shows the value of Rg_2d.

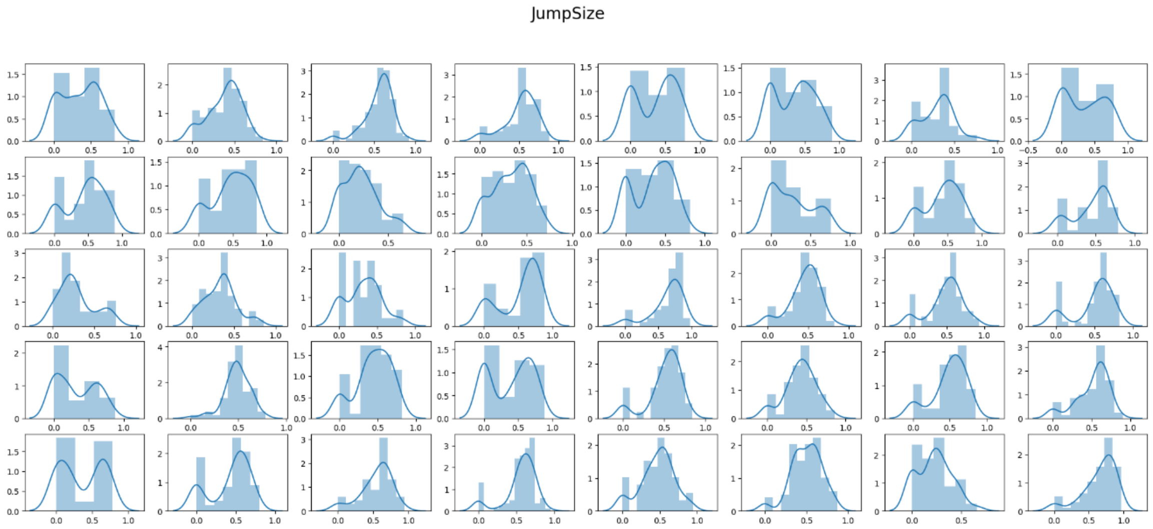

Figure S11. The density distribution of jump size, defined as the cosine similarity-based embedding distance between two consecutive publications, for randomly sampled 40 authors. Here the x-axis shows the cosine similarity-based embedding distance[12], and the y-axis shows the density.

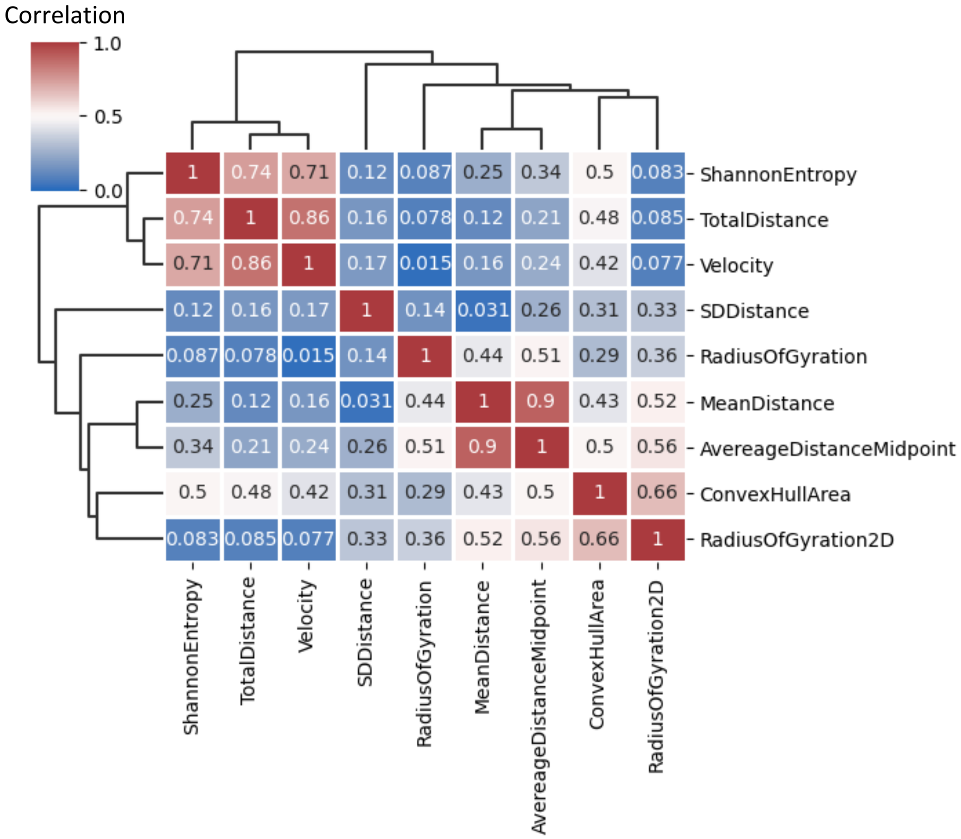

Figure S12. Heatmap of correlation between mobility measures. Here the total, mean, and SD distance are calculated as the sum, mean, and standard deviation of the cosine similarity-based embedding distance in individual trajectories. The velocity is calculated as the total distance divided by the year span of publications. The Shannon entropy[13] represents the diversity of individual mobility, i.e., the number of different transitions. The average distance to the midpoint is calculated as the average distance between the position of each publication and the center of mass. The convex hull area[14] is calculated as the volume of the convex hull of the individual trajectories in the 2-*d* space. Here we see two groups of measures; the first one—total distance, Shannon entropy, and velocity—we consider them largely correlated with the number of publications. The second group—SD distance, average distance to the midpoint, convex hull area, mean distance, and radius of gyration—captures the average amount of variability and the characteristic movements in the space.

Table S1. (A) The ten most similar periodicals to *PNAS*, a journal that broadly spans the biological, physical, and social sciences, based on the cosine similarity between embeddings. Multidisciplinary journals, such as *Science*, *Nature*, and some biological journals, are found on the list. (B) The ten most similar periodicals to *ICWSM (The International AAAI Conference on Web and Social Media)*, a conference that focused on online social media. Conferences such as *Web Science*, the *Web Conference*, *Conference on Online Social Networks*, and e-prints on arXiv in computer science are on the top list. (C) The ten most similar periodicals to *ASR (American Sociological Review)*. (D) The ten most similar periodicals to *JMLR (Journal of Machine Learning Research)*.

The ten most similar periodicals to PNAS

Periodicals	Similarity
**Science**	0.74
**Nature**	0.72
**PLOS Biology**	0.64
**Theoretical Ecology**	0.61
**Nature Communications**	0.61
**eLife**	0.60
**Current Biology**	0.60
**Cell**	0.60
**Science Advances**	0.59
**PLOS ONE**	0.59

The ten most similar periodicals to ICWSM

Periodicals	Similarity
**web science**	0.75
**arXiv: Social and Information Networks**	0.75
**ACM Transactions on The Web**	0.73
**the web conference**	0.71
**arXiv: Computers and society**	0.68
**web search and data mining**	0.67
**social informatics**	0.66
**conference on online social networks**	0.66
**ACM Sigweb Newsletter**	0.65
**ACM conference on hypertext**	0.65

The ten most similar periodicals to ASR	
**Periodicals**	**Similarity**
**Social Forces**	0.84
**Contemporary Sociology**	0.84
**American Journal of Sociology**	0.80
**Social Psychology Quarterly**	0.79
**Social Problems**	0.76
**Sociological Forum**	0.76
**Sociological Theory**	0.76
**Review of Sociology**	0.72
**Qualitative Sociology**	0.72
**Sociological Perspectives**	0.72

The ten most similar periodicals to JMLR	
**Periodicals**	**Similarity**
**neural information processing systems**	0.82
**international conference on artificial intelligence and statistics**	0.82
**arXiv: Machine Learning**	0.81
**international conference on machine learning**	0.80
**national conference on artificial intelligence**	0.73
**Machine Learning**	0.70
**siam international conference on data mining**	0.67
**arXiv: Learning**	0.64
**international conference on data mining**	0.61
**arXiv: Social and Information Networks**	0.61

Table S2: The linear regression model for academic success

	Average citations	75th percentile citations	Average (log(citations+1))
The standardized radius of gyration	-5.28***	-4.71***	-0.15***
	(0.39)	(0.24)	(0.01)
Humanities	-12.03**	-12.32***	-0.84***
	(5.04)	(4.69)	(0.21)
Medical and Health Sciences	0.10	1.13*	-0.06***
	(0.67)	(0.60)	(0.01)
Natural Sciences	13.05***	11.16***	0.32***
	(2.60)	(0.99)	(0.02)
Social Sciences	10.18***	12.42***	0.35***
	(0.77)	(0.67)	(0.02)
Asia	0.89	-0.32	0.05
	(1.65)	(1.60)	(0.06)
Europe	5.10***	3.53**	0.26***
	(1.74)	(1.59)	(0.06)
North America	11.20***	9.10***	0.37***
	(1.68)	(1.62)	(0.06)
Oceania	3.13*	2.19	0.25***
	(1.77)	(1.65)	(0.06)
South America	1.37	-1.35	0.02
	(2.02)	(1.69)	(0.07)
Female	0.41	-0.04	0.02
	(1.17)	(0.59)	(0.02)
Male	1.53***	0.86**	0.00
	(0.53)	(0.44)	(0.01)
Number of papers	0.01**	-0.00	0.00
	(0.00)	(0.00)	(0.00)
Academic age	0.82***	0.71***	0.02***
	(0.05)	(0.03)	(0.00)
R-squared	0.13	0.21	0.29
R-squared Adj.	0.13	0.20	0.28
N	10480	10480	10480

Robust standard errors in parentheses.
$* p < 0.05, ** p < 0.01, *** p < 0.001$

3. The embedding model with five dimensions

Table S3. (A) The ten most similar periodicals to PNAS. (B) The ten most similar periodicals to ICWSM (The International AAAI Conference on Web and Social Media).

The ten most similar periodicals to PNAS

Periodicals	Similarity
**The Plant Cell**	0.998
**Plant Methods**	0.998
**Plant Physiology**	0.997
**Nature**	0.996
**Mathematical Biosciences and Engineering**	0.995
**Journal of the Royal Society Interface**	0.991
**Plant Journal**	0.99
**Infectious Disease Modelling**	0.99
**arXiv: Populations and Evolution**	0.99
**Journal of Biological Dynamics**	0.989

The ten most similar periodicals to ICWSM

Periodicals	Similarity
**international conference on asian digital libraries**	0.998
**acm/ieee joint conference on digital libraries**	0.996
**International Journal on Digital Libraries**	0.995
**educational data mining**	0.995
**Information Visualization**	0.995
**User Modeling and User-adapted Interaction**	0.994
**Library Trends**	0.994
**international conference on social computing**	0.993
**international conference on information visualization theory and applications**	0.992
**international conference on user modeling, adaptation, and personalization**	0.992

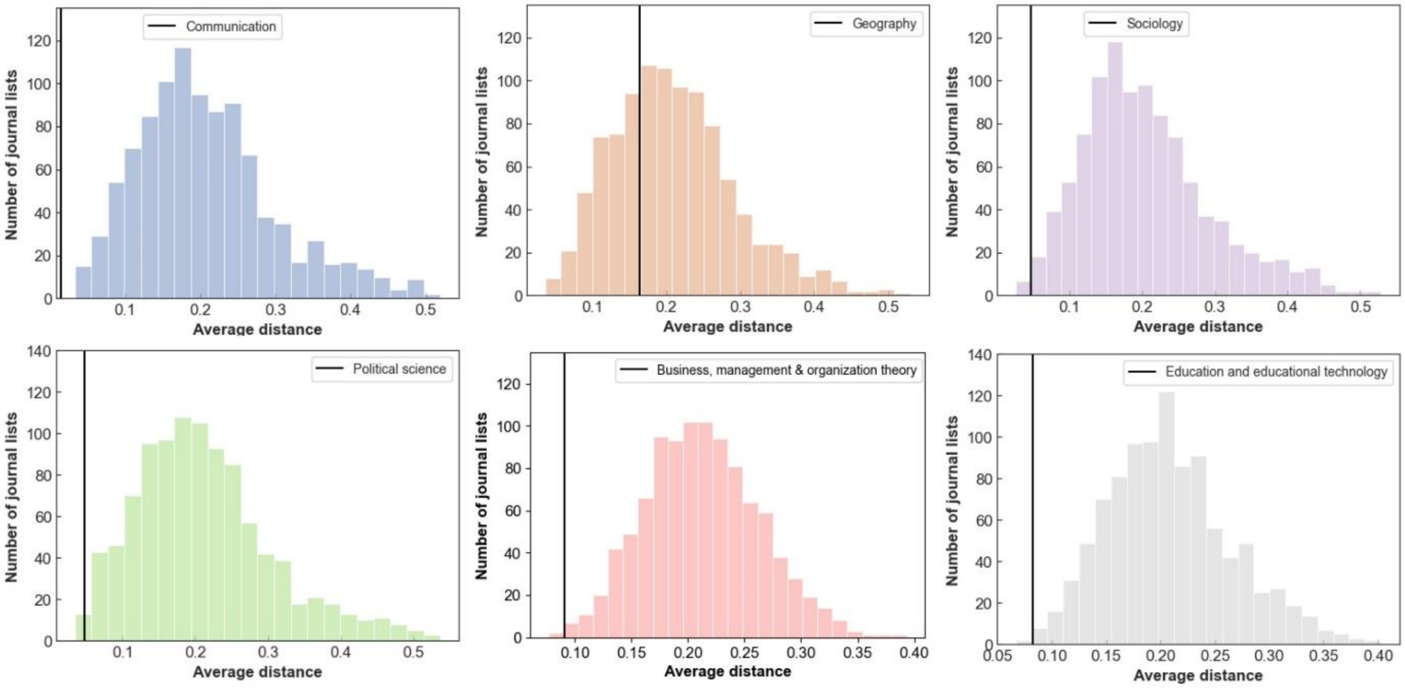

Figure S13. The average distance between journals within the same *subfield* (black line) and the distribution of the average distance between the same number of journals within the same *field*, based on 1000 randomly selected journal lists (color bars).

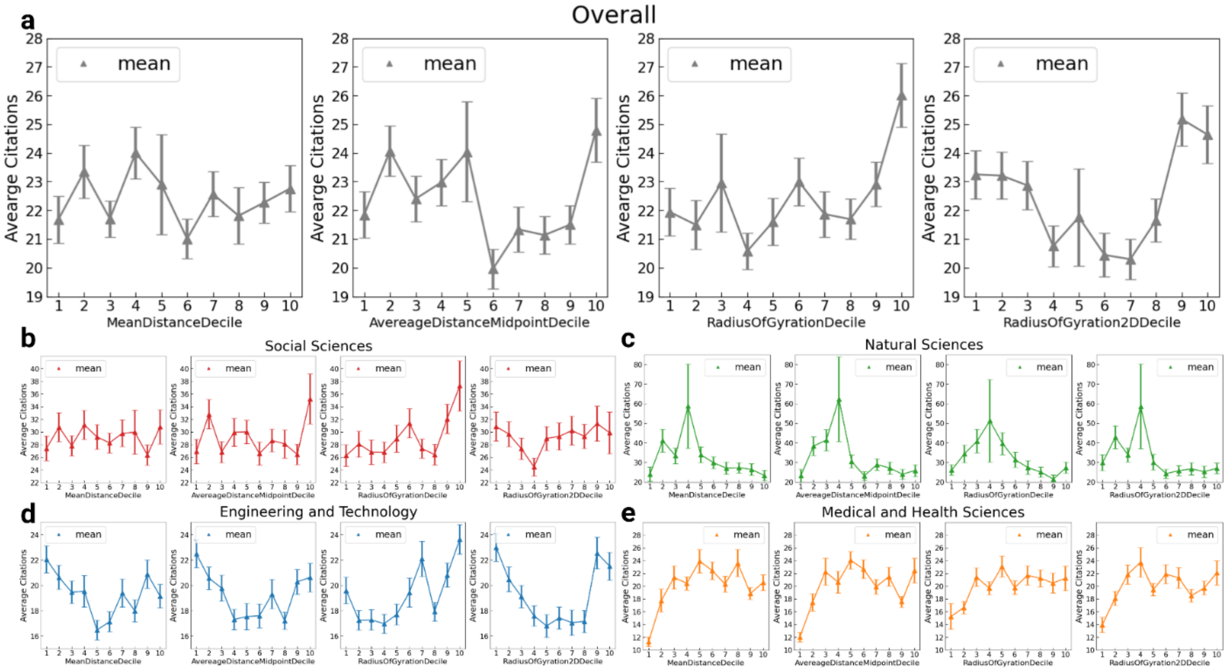

Figure S14. The average citations for authors in the deciles of four trajectory measures.

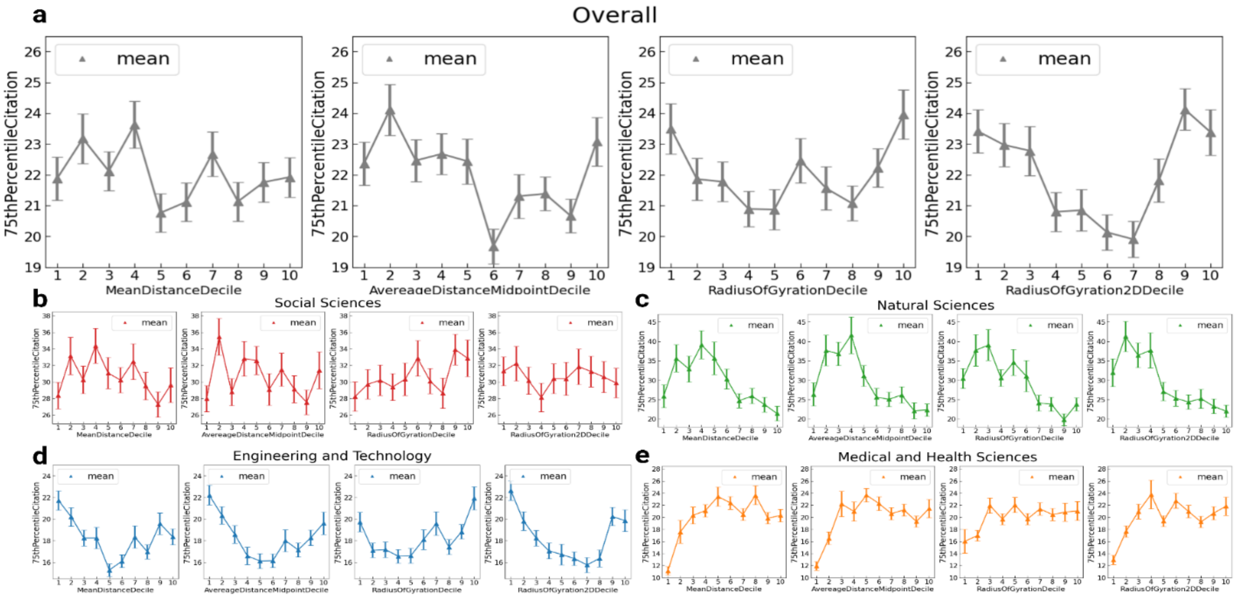

Figure S15. The 75th percentile citations for authors in the deciles of four trajectory measures.

References

1. Huang, J., Gates, A. J., Sinatra, R. & Barabási, A. L. Historical comparison of gender inequality in scientific careers across countries and disciplines. *Proc. Natl. Acad. Sci. U. S. A.* **117**, 4609–4616 (2020).
2. Jadidi, M., Karimi, F., Lietz, H. & Wagner, C. Gender disparities in science? Dropout, productivity, collaborations and success of male and female computer scientists. *Adv. Complex Syst.* **21**, (2018).
3. Holman, L., Stuart-Fox, D. & Hauser, C. E. The gender gap in science: How long until women are equally represented? *PLoS Biol.* **16**, e2004956 (2018).
4. Karimi, F., Wagner, C., Lemmerich, F., Jadidi, M. & Strohmaier, M. Inferring Gender from Names on the Web: A Comparative Evaluation of Gender Detection Methods. *WWW 2016 Companion - Proc. 25th Int. Conf. World Wide Web* 53–54 (2016) doi:10.1145/2872518.2889385.
5. Larivière, V., Ni, C., Gingras, Y., Cronin, B. & Sugimoto, C. R. Global gender disparities in science. *Nature* **504**, 211–213 (2013).
6. Van Der Maaten, L. & Hinton, G. Visualizing data using t-SNE. *J. Mach. Learn. Res.* **9**, 2579–2625 (2008).
7. Wang, Y., Huang, H., Rudin, C. & Shaposhnik, Y. Understanding How Dimension Reduction Tools Work: An Empirical Approach to Deciphering t-SNE, UMAP, TriMap, and PaCMAP for Data Visualization. *J. Mach. Learn. Res.* **22**, 1–73 (2021).
8. McInnes, L., Healy, J. & Melville, J. UMAP: Uniform Manifold Approximation and Projection for Dimension Reduction. *arXiv* (2018) doi:10.48550/arxiv.1802.03426.
9. Jollife, I. T. & Cadima, J. Principal component analysis: a review and recent developments. *Philos. Trans. R. Soc. A* **374**, (2016).
10. Macqueen, J. Some Methods for Classification and Analysis of MultiVariate Observations. *Proc Berkeley Symp. Math. Stat. Probab.* **5**, 281–297 (1967).
11. Gates, A. J., Wood, I. B., Hetrick, W. P. & Ahn, Y. Y. Element-centric clustering comparison unifies overlaps and hierarchy. *Sci. Rep.* **9**, 1–13 (2019).
12. Murray, D. *et al.* Unsupervised embedding of trajectories captures the latent structure of scientific migration. *Proc. Natl. Acad. Sci.* **120**, (2023).
13. Shannon, C. E. A Mathematical Theory of Communication. *Bell Syst. Tech. J.* **27**, 379–423 (1948).
14. Bohr, J., Markvorsen, S. & Raffaelli, M. Newson's challenge and the volume of certain convex hulls. *arXiv* at https://doi.org/10.48550/arxiv.1805.11335 (2018).
